# A Case Report of Steroid-Induced Angioedema and Urticaria

**DOI:** 10.7759/cureus.46515

**Published:** 2023-10-05

**Authors:** Leo Wan, John Thomas, Audrey Yan, Jake Mann, Anthony Szema

**Affiliations:** 1 Medical School, West Virginia School of Osteopathic Medicine, Lewisburg, USA; 2 Department of Medicine, West Virginia School of Osteopathic Medicine, Lewisburg, USA; 3 Department of Occupational Medicine, Epidemiology, and Prevention, Donald and Barbara Zucker School of Medicine at Hofstra/Northwell, Hempstead, USA

**Keywords:** clobetasol propionate, prednisolone, dermatology, urticaria, patch test, type iv hypersensitivity, steroid allergy, angioedema

## Abstract

Physicians regularly use corticosteroids to treat various conditions, attributing their anti-inflammatory and immunosuppressive properties. Cases of allergic sensitivity reactions and dermatitis induced by corticosteroids are relatively uncommon. We present a case regarding an 81-year-old male with a history of actinic keratosis, atopic dermatitis, and psoriasis, who experienced a Type I hypersensitivity reaction with facial angioedema and urticaria on his axilla, torso, and popliteal fossa that developed after treatment with oral prednisolone. This episode also exacerbated his previously diagnosed psoriasis. To treat psoriasis, a dermatologist prescribed clobetasol topical ointment, which did not alleviate the symptoms; instead, it only exacerbated the rash, and he was subsequently referred for corticosteroid allergy testing. North American 85 Comprehensive Series patch testing revealed a positive test for various classes of steroids, including clobetasol-17-propionate, budesonide, and dexamethasone, thus proving a T cell-mediated allergy to corticosteroids.

## Introduction

Corticosteroids are steroid hormone drugs used to treat a vast array of conditions. Given their anti-inflammatory and immunosuppressive properties [1], the drugs can treat allergic disorders, asthma, and autoimmune diseases. Multiple forms of these drugs exist to treat this wide range of conditions, including oral, injection, inhalation, and topical. Corticosteroid allergies are rare but can produce mild rash to life-threatening symptoms if mounted [2].

Corticosteroid allergies can be grouped into Type I (immediate reaction) or Type IV (non-immediate reaction), differing in prevalence, symptoms, and severity. An allergic response within one hour of administration is considered Type I, while symptoms presenting after the hour are characterized as Type IV. Type I is rare (0.3-0.5%) [3], immunoglobulin mediated, and causes acute symptoms such as anaphylaxis [2]. Type IV is more prevalent in the United States (4.6%), is T-cell mediated, and produces more chronic symptoms. This reaction is seen chiefly with topical/skin corticosteroids and usually manifests in allergic contact dermatitis [4].

Topical corticosteroids are essential in managing dermatological conditions such as eczema and psoriasis due to their anti-inflammatory and immunosuppressive effects. In addition, the application directly to the skin allows for the observation of treatment effectiveness and subsequent dosage and frequency adjustments [5]. Topical corticosteroids are divided into seven classes based on potency, with class I being the most potent and commonly used for sites with thick epidermis [6].

## Case presentation

**Figure 1:**
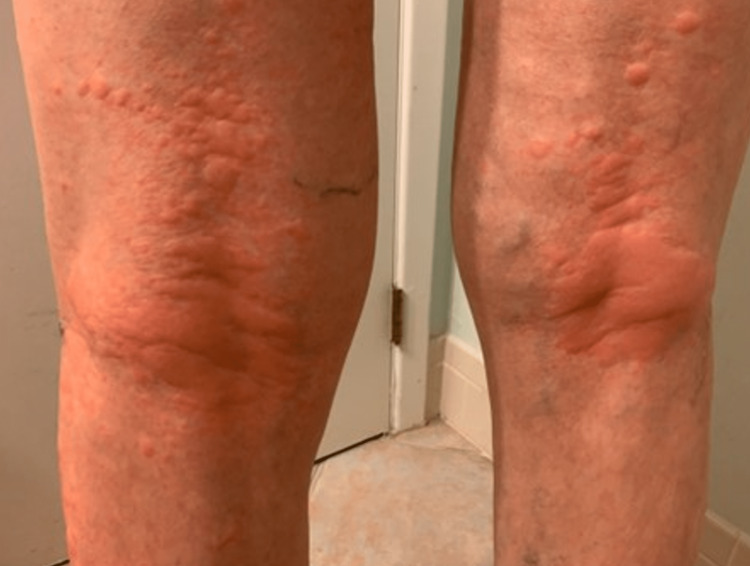
Urticaria on bilateral popliteal fossa

**Figure 2:**
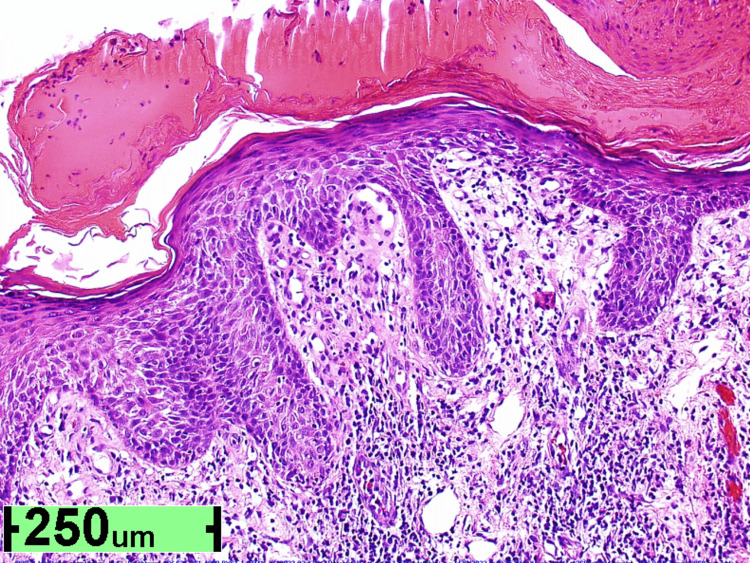
Shaved skin biopsy (right proximal posterior upper arm) Epidermal spongiosis with edema and dilated tortuous capillaries widening dermal papillae and mixed lichenoid and perivascular infiltrate of eosinophils and lymphocytes. PAS stains tiny plasma crusts atop the squamous epidermis yet fails to reveal fungal hyphae. Deeper levels fail to reveal signs of vasculitis, mites of scabies, or signs of bullous pemphigoid. This histology finding hints at psoriasis, now complicated by a superimposed allergic process, such as allergic contact dermatitis or drug eruption. PAS: periodic acid–Schiff

An 81-year-old male with a history of actinic keratosis, eczema, and psoriasis developed angioedema on his face and lips, along with pruritic wheals on the torso, axilla, and popliteal fossa (Figure 1) bilaterally after taking 20 mg prednisolone once a day for five days, which is a steroid used to treat acute bronchitis. The itchy wheals developed on his torso and bilateral popliteal fossa after five hours of the first dose of prednisolone, and the dermatologist prescribed topical clobetasol 0.05% ointment due to history of treatment with steroids earlier, considering a chronic case of eczema and psoriasis. However, the treatment ultimately exacerbated the rash on the left axilla, causing more erythema and pruritis. In response, a shave biopsy was done on the right proximal posterior upper arm and left anterior proximal thigh; substantial spongiotic changes closely resemble allergic contact dermatitis despite clinical pictures resembling psoriasis (Figure 2). 

**Figure 3:**
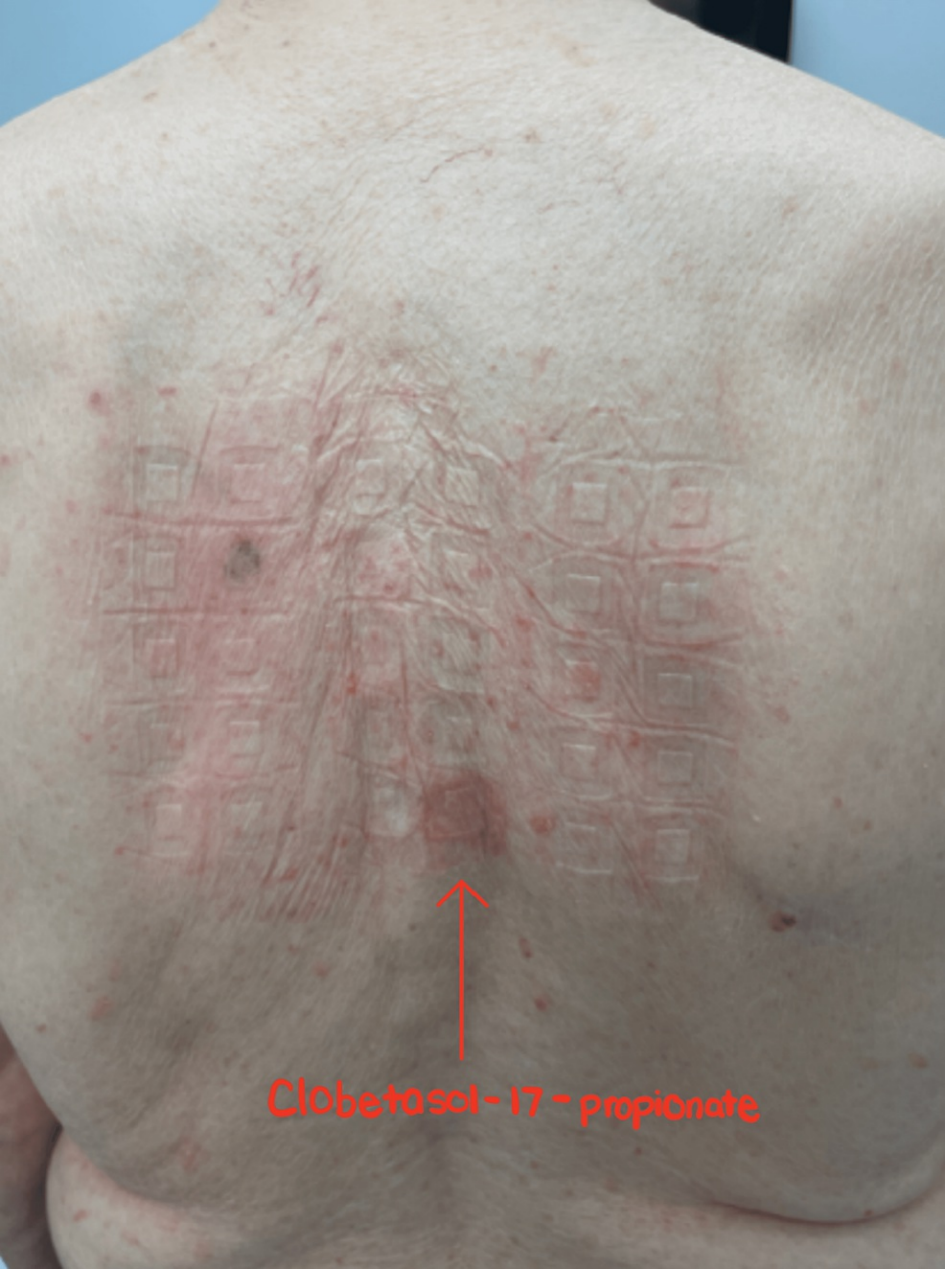
Positive patch test for #11: clobetasol-17-propionate

**Figure 4:**
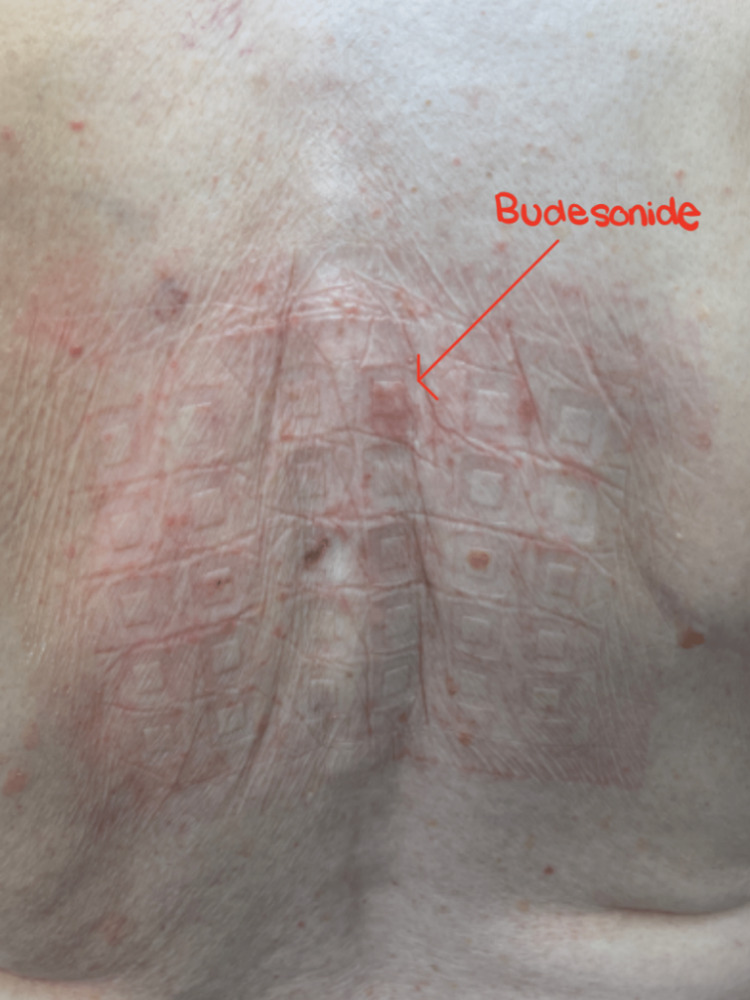
Positive patch test for #45: budesonide

**Figure 5:**
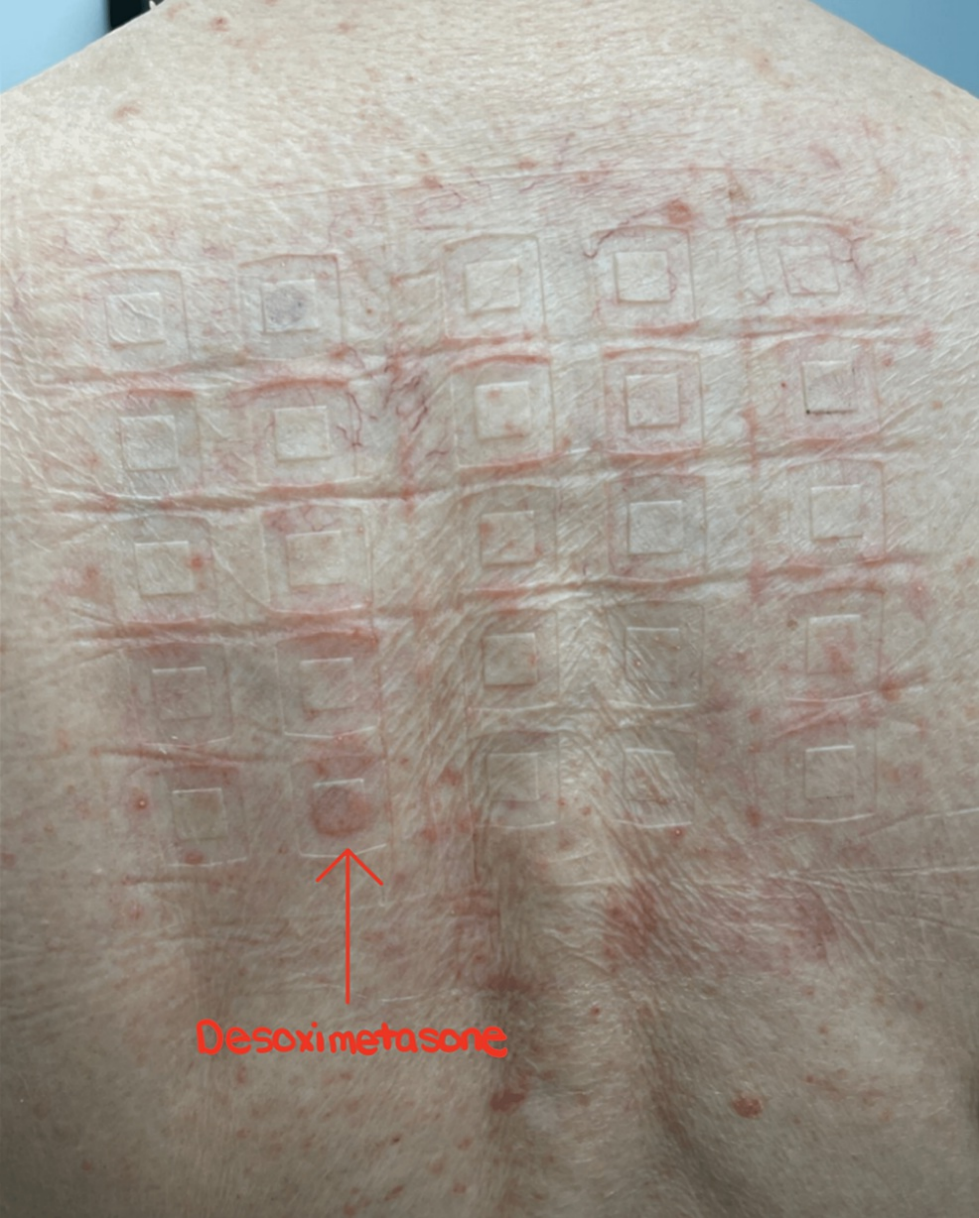
Positive test for #61: desoximetasone

The dermatologist ultimately referred the patient to an allergist specialist, who initiated the North American 85 Comprehensive Series patch test. After a series of follow-ups, we removed his patch and saw well-demarcated erythematous rashes greater than 5 mm for allergen number 11: clobetasol-17-propionate (Figure 3), number 45: budesonide (Figure 4), number 61: desoximetasone (Figure 5), indicating allergic contact dermatitis to three different types of steroids. The patient was instructed to immediately discontinue the offending agents.

## Discussion

Angioedema is a mast-cell mediated immune response characterized by swelling under the skin, either accompanied with or without urticaria. In this patient’s case, urticaria described as pruritic wheals were present. In response to the corticosteroids, immunoglobulin E (IgE) antibodies present on the surface of mast cells bind to the steroid allergen, releasing histamines and triggering an immune response present as angioedema and urticaria. Steroid-induced allergic reactions are rare, with the overall prevalence of type I IgE-mediated steroid hypersensitivity being about 0.3-0.5% [2].

Corticosteroids are grouped under the Coopman classification based on chemical structure: Groups A, B, C, and D: D1 and D2. Our 81-year-old male patient ultimately presented with allergic reactions to all groups of steroids except for D2. Prednisone belongs to Group A, budesonide belongs to Group B, desoximetasone belongs to Group C, and clobetasol-17-propionate belongs to Group D1 [7]. Cross-reactions often occur between compounds of similar structure but can also exist between those of different classes. Clinicians should be wary and closely monitor for any adverse reactions if deciding to prescribe another steroid within any of these four classes. While no adverse response was seen with Group D2 steroids, sensitivity to these steroids may still occur. Group D2 steroids have been known to cross-react with budesonide [7].

## Conclusions

This study demonstrated that it is crucial to consider patch testing for corticosteroids to detect allergic hypersensitivity in patients present with angioedema and urticaria after a course of steroid treatment. The development of worsening symptoms after initiating corticosteroids should alert physicians to consider the possibility of an allergic hypersensitivity reaction to various classes of steroids. Furthermore, the patch test can confirm an allergy to previously utilized corticosteroids, and identify sensitivity to unencountered corticosteroids in order to minimize medical errors.
